# Dephosphorylation in nuclear reassembly after mitosis

**DOI:** 10.3389/fcell.2022.1012768

**Published:** 2022-10-04

**Authors:** Vincent Archambault, Jingjing Li, Virginie Emond-Fraser, Myreille Larouche

**Affiliations:** ^1^ Institute for Research in Immunology and Cancer, Université de Montréal, Montréal, QC, Canada; ^2^ Département de Biochimie et Médecine Moléculaire, Université de Montréal, Montréal, QC, Canada

**Keywords:** mitosis, mitotic exit, nucleus, nuclear envelope, phosphatase, chromosome decondensation, kinetochore disassembly, nucleolus

## Abstract

In most animal cell types, the interphase nucleus is largely disassembled during mitotic entry. The nuclear envelope breaks down and chromosomes are compacted into separated masses. Chromatin organization is also mostly lost and kinetochores assemble on centromeres. Mitotic protein kinases play several roles in inducing these transformations by phosphorylating multiple effector proteins. In many of these events, the mechanistic consequences of phosphorylation have been characterized. In comparison, how the nucleus reassembles at the end of mitosis is less well understood in mechanistic terms. In recent years, much progress has been made in deciphering how dephosphorylation of several effector proteins promotes nuclear envelope reassembly, chromosome decondensation, kinetochore disassembly and interphase chromatin organization. The precise roles of protein phosphatases in this process, in particular of the PP1 and PP2A groups, are emerging. Moreover, how these enzymes are temporally and spatially regulated to ensure that nuclear reassembly progresses in a coordinated manner has been partly uncovered. This review provides a global view of nuclear reassembly with a focus on the roles of dephosphorylation events. It also identifies important open questions and proposes hypotheses.

## Introduction

### Mitosis: Disassembling one to reassemble two

**FIGURE 1:**
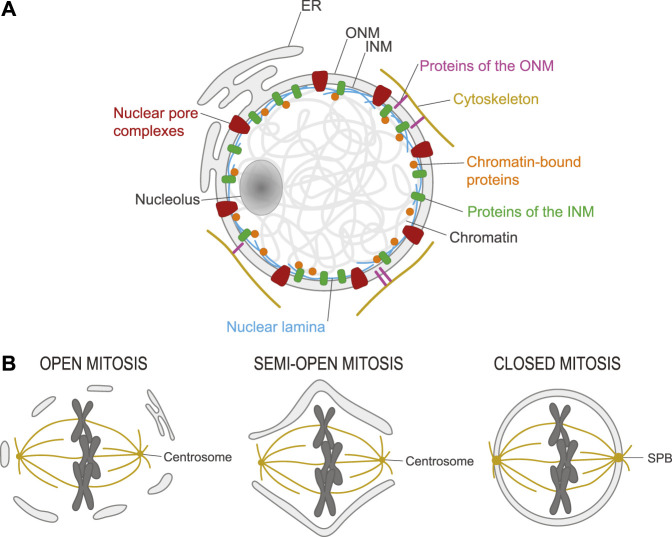
Structure of the nucleus and its transformation in mitosis. **(A)**. Chromosomes are contained in a nucleus in eukaryotic cells. This nucleus is delineated by a nuclear envelope (NE) composed of the inner nuclear membrane (INM) and the outer nuclear membrane (ONM) which is continuous with the endoplasmic reticulum (ER). Nuclear pore complexes (red) control exchanges between the nucleus and the cytoplasm. The nuclear lamina (blue) confers rigidity to the nucleus and interacts with chromatin-bound proteins (orange) and transmembrane proteins of the INM (green). Some transmembrane proteins of the ONM (pink) interact with elements of the cytoskeleton (yellow). The nucleolus is a specialized intranuclear structure where ribosomes are synthesized. **(B)**. Left) Open mitosis. In most animal cells, the NE breaks down in mitosis, allowing microtubules of the spindle emanating from centrosomes to connect with and segregate chromosomes. Right) Closed mitosis. In most fungi, the NE does not break down in mitosis. Instead, spindle pole bodies (SPB) are embedded in the NE and assemble a spindle that spans the nucleus. Center) Semi-open mitosis. In some species, the NE does not completely break down but fenestrates at the poles to allow microtubules of the spindle emanating from centrosomes to reach chromosomes.

In eukaryotes, chromosomes are encapsulated in a nucleus delineated by the nuclear envelope (NE) which is composed of two membranes (Figure 1A) (Alberts et al., 2015). The Inner Nuclear Membrane (INM) contains several integral proteins that make contacts with proteins attached to chromatin. Conversely, the Outer Nuclear Membrane (ONM) contains proteins that make contacts with proteins of the cytoskeleton. In addition, the ONM is continuous with the Endoplasmic Reticulum (ER), which contributes to several cellular functions including protein synthesis (Ungricht and Kutay, 2017; Carlton et al., 2020). This sequestration of chromatin from the cytoplasm and the organelles it contains effectively separates transcription from translation (Alberts et al., 2015). Exchanges between the nucleus and the cytoplasm occur via nuclear pores, which are gated by large protein assemblies named Nuclear Pore Complexes (NPCs) (Wente and Rout, 2010). Inside the nucleus, chromatin is organized into various domains which respond dynamically to demands in transcription, to DNA damage/repair and to mechanical forces (Zheng and Xie, 2019; Dupont and Wickstrom, 2022; Stanic and Mekhail, 2022). The physical separation between the nucleus and the cytoplasm also facilitates the regulation of signaling and cell cycle transitions by the sequestration and concentration of proteins and enzymatic activities (Pintard and Archambault, 2018).

Mitosis is the process by which the nucleus divides (Morgan, 2007). In most cell types, it is coupled to cytokinesis, the division of the entire cell. In animal species, the NE is broken in mitosis, to allow chromosome attachment and segregation on a spindle of microtubules (MTs) that is organized in a bipolar manner by two cytoplasmic centrosomes (open mitosis; Figure 1B) (Guttinger et al., 2009). In yeast and many other fungi, the NE does not break down and a bipolar spindle of MTs is positioned across the mitotic nucleus by two Spindle Pole Bodies (SPBs) inserted into the NE (closed mitosis; Figure 1B) (Kilmartin, 2014). In some animal species including *Drosophila melanogaster*, the NE does not break down completely, but it fenestrates at least enough to allow the spindle to connect chromosomes and centrosomes (semi-open mitosis; Figure 1B) (Sazer et al., 2014). In all cases, chromosomes become condensed and separated from each other to facilitate their sorting in mitosis; they also assemble kinetochores (KTs) onto their centromeres, to allow chromosome attachment to spindle MTs (Morgan, 2007).

### Reversible phosphorylation cycles drive mitotic progression

**FIGURE 2:**
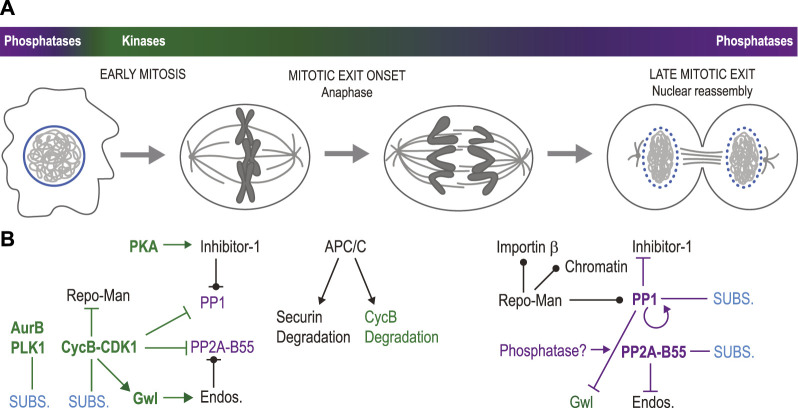
Elements of temporal regulation in nuclear reassembly. **(A)**. During mitotic entry, mitotic kinases become more active while some mitotic phosphatases become less active, causing NEBD, chromosome condensation and spindle assembly. Mitotic exit begins with anaphase onset, followed by nuclear reassembly. During mitotic exit, mitotic kinases become generally less active while phosphatases regain their full activity. **(B)**. Molecular networks at play in the transitions displayed in panel **(A)**. At the beginning of mitosis (left), Cyclin B-CDK1, Aurora B, PLK1 and other mitotic kinases phosphorylate multiple substrates (SUBS.) leading to the disassembly of the interphase nucleus. Concomitantly, PP1 and PP2A-B55 are inhibited. PP1 phosphorylation by Cyclin B-CDK1 inhibits its activity. PP1 is also inhibited by Inhibitor-1 phosphorylated by PKA. Repo-Man, the PP1 regulatory subunit, is phosphorylated at multiple CDK sites that inhibit its interactions with PP1, its targeting to chromatin and its interaction with Importin *β*. CDK1-activated Gwl kinase phosphorylates Endosulfine proteins (Endos.) which then inhibit PP2A-B55. CDK1 also inhibits PP2A-B55 complex formation by phosphorylating PP2A-C. At the onset of mitotic exit (center), anaphase is triggered by the activation of the APC/C, which promotes the degradation of Securin, allowing sister chromatid separation. Cyclin B is also largely degraded at this time in an APC/C-dependent manner. At later stages of mitotic exit (right), PP1 dephosphorylates itself, thereby becoming active. It also dephosphorylates Inhibitor-1 (relieving inhibition), Repo-Man (allowing it to interact with PP1, chromatin and Importin β) and Gwl (inactivating it). Endosulfines dephosphorylation by PP2A-B55 may then be completed, relieving PP2A-B55 inhibition. At that stage, PP1 and PP2A-B55 can dephosphorylate several proteins to promote nuclear reassembly. The phosphatase responsible for PP2A-C dephosphorylation is unknown. Green names: kinases; purple names: phosphatases; bold names: active enzymes; pointed arrows: activation; blunt-ended arrows: inhibition: circle-ended arrows: interaction; green connections: phosphorylation; purple connections: dephosphorylation.

Mitotic entry is triggered by a strong wave of activity of serine/threonine kinases including Cyclin-Dependent Kinases (CDKs; particularly CDK1 and CDK2), Polo-Like Kinase 1 (PLK1) and Aurora kinases (Aurora A and Aurora B) (Morgan, 2007; Carmena et al., 2009; Lindqvist et al., 2009; Heim et al., 2017; Pintard and Archambault, 2018) (Figure 2A). Mitosis requires the dissociation of some protein complexes and the formation of others, ultimately resulting in molecular events that shape intracellular structures. Phosphorylation often disrupts protein interactions by modifying the charge or the structure of binding sites (Nishi et al., 2011; Nishi et al., 2014). In species with open mitosis, the burst in kinase activity during mitotic entry seems perfectly poised to promote nuclear envelope breakdown (NEBD) and the dismantling of other nuclear structures, the integrity of which depends on numerous protein interactions. Other structures, like centrosomes or kinetochores, need to assemble in mitosis and take advantage of phosphorylation events that promote protein interactions (Hara and Fukagawa, 2018; Ong et al., 2020). The transition from mitosis to interphase (mitotic exit) requires the inactivation of many mitotic phosphoproteins. While several cell cycle regulatory proteins such as cyclins are degraded in a ubiquitin-dependent manner to enable cell cycle transitions, relatively few effector proteins are (Min and Lindon, 2012). Instead, in most cases, effector proteins that are phosphorylated in mitosis become dephosphorylated as the cell exits mitosis (Holder et al., 2019).

Phosphoproteomic studies have profiled changes in global protein phosphorylation as cells exit mitosis. Studies with HeLa cells found hundreds of sites that are dephosphorylated during mitotic exit, many of them in structural nuclear proteins (Malik et al., 2009; McCloy et al., 2015; Holder et al., 2020). Fewer sites were found to increase in phosphorylation during mitotic exit. In the budding yeast, however, a recent study found similar numbers of sites hyperphosphorylated and hypophosphorylated during mitotic exit (Touati et al., 2018). This difference in profiles between yeast and human cells could reflect a fundamental divergence in the molecular regulation of mitotic exit in open vs. closed mitosis. Indeed, the events of mitotic exit in *S. cerevisiae* are largely controlled by the Cdc Fourteen Early Anaphase Release (FEAR) network and by the Mitotic Exit Network (MEN), which involve several kinases (Rock and Amon, 2009; Baro et al., 2017). These signaling cascades culminate with the activation of the Cdc14 phosphatase, which is responsible for the dephosphorylation of several CDK targets. Signaling akin to FEAR or MEN, or Cdc14 orthologs, does not appear to play major roles in mitotic exit in humans (Mocciaro and Schiebel, 2010; Wurzenberger and Gerlich, 2011).

Nuclear Reassembly (NR) at the end of open mitosis requires the dephosphorylation of several targets. At least two groups of serine/threonine phosphatases of the Phospho Protein Phosphatase (PPP) family play essential roles in the transition from mitosis to interphase in animals: Protein Phosphatase 1 (PP1) and Protein Phosphatase 2 A (PP2A) (Shi, 2009; Holder et al., 2019). In humans, there are three versions of the catalytic subunit of PP1 that are produced by distinct genes: PP1α, PP1β, and PP1γ. These can interact with over 200 regulatory subunits to form holoenzymes (Bollen et al., 2010; Moura and Conde, 2019). PP1 uses several regulatory subunits adaptors including Repo-Man, AKAP149, PNUTS and Sds22 to promote NR in multiple ways (Moura and Conde, 2019). The catalytic PP2A protein (PP2A-C) comes in two isoforms: PP2ACα and PP2ACβ that assemble mostly trimers with a structural subunit (PP2A-A) and one of many interchangeable regulatory subunits of various types (PP2A-B) (Fowle et al., 2019; Moura and Conde, 2019). To execute their functions in mitotic exit, PP1 and PP2A associate with specific regulatory subunits. PP2A in complex with its B55 regulatory subunits (PP2A-B55) efficiently targets sites phosphorylated by CDKs (Agostinis et al., 1992; Ferrigno et al., 1993; Mayer-Jaekel et al., 1994; Castilho et al., 2009; Mochida et al., 2009; Amin et al., 2021). PP2A-B55 enzymes also dephosphorylate sites phosphorylated by other mitotic kinases (Amin et al., 2021). Depletion of B55α in human cells results in a delay of late events of mitotic exit including nuclear envelope reformation (NER) and chromosome decondensation (Schmitz et al., 2010). Other members of the PPP family, including PP4 and PP6 are also emerging as regulators of mitosis (Shi, 2009).

## Mechanistic aspects of dephosphorylation in nuclear reassembly

Once chromosomes have segregated in anaphase, the nucleus is reassembled through the coordination of NER, chromosome decondensation, chromatin organization and kinetochore disassembly. Many excellent reviews have covered in extensive detail the mechanisms governing the transformations of particular nuclear structures through the mitotic cycle. Here, we provide a more global mechanistic view with a particular focus on the roles of dephosphorylation in promoting molecular events in the reconstruction of the nucleus during mitotic exit. We draw lessons from findings in various species; however, we concentrate on how things occur in mammalian cells and the text refers to mammalian proteins unless otherwise indicated. While we do not intend to exhaustively describe in detail the regulation of all dephosphorylation substrates implicated in nuclear reassembly, a sampling of some of the main and best-characterized targets is provided in Table 1.

**TABLE 1:** Protein dephosphorylation events with roles in post-mitotic nuclear reassembly. The list contains a selection of known or probable substrates whose dephosphorylation participates in nuclear reassembly events. Elements that are less certain in view of the current literature are followed by a question mark. ND: Not determined. Multisite: many phosphorylation sites were identified and may contribute to the regulation.

Substrate	Kinase(s)	Site(s)	Phosphatase(s)	Proposed function dephosphorylation	Selected reference(s)
Joining chromosomes					
BAF	VRKs	Thr2? Thr3 Ser4	PP2A PP4	Enhances DNA binding	Nichols et al. (2006), Asencio et al. (2012)
					Zhuang et al. (2014)
					Mehsen et al. (2018)
					Torras-Llort et al. (2020)
Ki-67	CDK1	Multisite	PP1?	Promotes chromosome clustering	Booth et al. (2014)
					Yamazaki et al. (2022)
Membrane recruitment					
LBR	SRPK1 CDK1	RS motifs Ser71, Ser86	PP1 PP2A?	Promotes LBR multimers, Enhances chromatin binding	Takano et al. (2004), Tseng and Chen (2011)
					Mimura et al. (2016)
Emerin	ND	Multisite?	ND	Promotes binding to BAF	Hirano et al. (2005)
Man1	ND	Multisite?	ND	Promotes binding to BAF	Hirano et al. (2009)
Lamina assembly					
Lamin B1/B2	CDK1 PKC	Multisite	PP1-AKAP149? PP2A?	Polymerization of nuclear lamins, lamina reassembly	Peter et al. (1990), Peter et al. (1991)
					Goss et al. (1994)
					Thompson et al. (1997)
					Steen et al. (2000)
					Kuga et al. (2010)
					Kauko et al. (2020)
Lamin A/C	CDK1 PKC	Ser22 + Multisite	PP1γ-Repo-Man PP2A?	Polymerization of nuclear lamins, lamina reassembly	Heald and McKeon (1990)
					Eggert et al. (1993)
					Kauko et al. (2020)
					Moriuchi and Hirose (2021)
NPC assembly					
Nup53	CDK1PLK1	Multisite	ND	Promotes interactions with multiple Nups within NPC	McCloy et al. (2015), Linder et al. (2017)
					Martino et al. (2017)
Nup98	CDK1 PLK1NEKs	Multisite	ND	Promotes interactions with multiple Nups within NPC	Laurell et al. (2011)
					Linder et al. (2017)
Nup107	CDK1	Multisite	PP2A-B55	Promotes interaction with other Nups?	McCloy et al. (2015)
					Cundell et al. (2016)
Nup153	ND	Ser257	PP2A-B55 PP1γ-Repo-Man?	Promotes recruitment to NE/chromatin in telophase	Cundell et al. (2016)
NCD1	ND	Thr414	PP2A-B55	Promotes interaction with other Nups?	Cundell et al. (2016)
NE sealing					
CHMP7	CDK1	Ser3 Ser441	PP1?	Restores interaction with LEM2 for ESCRT-III recruitment to membranes	Asencio et al. (2012) Gatta et al. (2021)
LEM2	ND	Multisite? (disordered region)	PP1?	Restores ability to interact with MTs and oligomerize	Asencio et al. (2012)
					von Appen et al. (2020)
Chromosome decondensation					
Histone H3	Haspin Aurora B	Thr3 Ser10	PP1γ-Repo-Man	Promotes nucleosome acetylation and Interactions	Qian et al. (2011)
CAP-H (Condensin I)	Aurora B	Ser70	ND	Promotes dissociation from chromosomes?	Tada et al. (2011)
CAP-G (Condensin I)	CDK1	Thr308 Thr332	ND	Promotes dissociation from chromosomes?	Murphy and Sarge (2008)
CAP-H2 (Condensin II)	PLK1	Ser288	ND	Promotes dissociation from chromosomes?	Kagami et al. (2017)
CAP-D3 (Condensin II)	CDK1	Thr1415	ND	Prevents PLK1 binding and its phosphorylation of Condensin II?	Abe et al. (2011)
Chromatin & Nucleolus organization					
Histone H3	Aurora B	Ser10	PP1γ-Repo-Man	Promotes binding to HP1	Hirota et al. (2005), Vagnarelli et al. (2011)
Nucleophosmin (B23)	CKII CDK1	Ser125 Thr199 + Multisite	PP1-Ki-67? PP1β,γ-RRP1B?	Promotes nucleolar reassembly?	Okuwaki et al. (2002)
					Negi and Olsen (2006)
					Chamousset et al. (2010)
					Booth et al. (2014)
					Yamazaki et al. (2022)
Kinetochore disassembly					
Dsn1	Aurora B	Ser100 Ser109	PP1-Sds22? PP1-Repo-Man?	Promotes dissociation from CENP-C?	Welburn et al. (2010), Yang et al. (2008)
					Wurzenberger et al. (2012)
					Kim and Yu (2015)
					Rago et al. (2015)
CENP-C	CDK1	Thr734	ND	Promotes dissociation from CENP-A?	Watanabe et al. (2019)
CENP-T	CDK1	Thr11 Thr85	ND	Promotes dissociation from Ndc80 complex?	Nishino et al. (2013)
					Rago et al. (2015)
		Ser201	ND	Promotes dissociation from Mis12 complex?	Huis In 't Veld et al. (2016)

### Shaping a single nucleus

**FIGURE 3:**
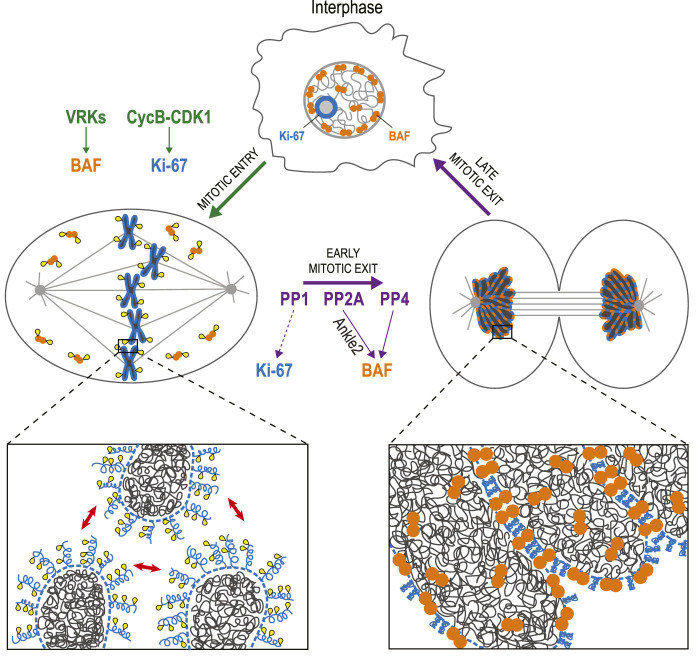
Dephosphorylation of BAF and Ki-67 promotes the assembly of a single nucleus. In interphase, BAF (orange) is mostly restricted to the nuclear periphery, while Ki-67 (blue) localizes to nucleoli. During mitotic entry, VRKs phosphorylate BAF, inducing its dissociation from chromatin. Cyclin B-CDK1 phosphorylates Ki-67, which becomes concentrated in the perichromosomal compartment (dotted line) and mediates a repulsion between chromosomes (red arrows). During mitotic exit, BAF is dephosphorylated by PP2A (assisted by Ankle2) and PP4. As a result, BAF dimers bind DNA, linking chromosomes together into a single mass during telophase. Upon dephosphorylation, possibly by PP1, Ki-67 also contributes to the cohesion of a tight chromatin mass from which cytoplasmic components are excluded while the nucleus reassembles. Yellow: phosphate groups.

Mitosis requires means to sort individual chromosomes, and to repackage them into a single nucleus (Figure 3). Ki-67 is a protein that has been used for decades as a cellular marker of cell proliferation (Remnant et al., 2021). During interphase, it localizes to nucleoli, and during mitosis, it localizes around chromosomes, in the perichromosomal compartment (Booth et al., 2014). This scaffold surrounding mitotic chromosomes is still incompletely characterized but Ki-67 is required for its recruitment of several other proteins (Booth et al., 2014). Ki-67 was recently shown to act as a surfactant that coats chromosomes in mitosis and promotes their dispersal (Booth et al., 2014; Cuylen et al., 2016; Stamatiou and Vagnarelli, 2021). This repulsion is mediated by the unstructured, brush-like organization of Ki-67 in the early stages of mitosis (Cuylen et al., 2016). It was recently shown that this configuration is induced by multisite phosphorylation of Ki-67 that creates alternative charge blocks promoting liquid-liquid phase separation (LLPS) (Yamazaki et al., 2022). During anaphase, the brush-like structures collapse as Ki-67 switches to a state that promotes chromosome clustering, thereby excluding the cytoplasmic content during NR (Cuylen-Haering et al., 2020). This transition presumably requires a dephosphorylation of Ki-67 that has not been characterized. Ki-67 interacts with PP1 but this interaction does not appear to be required for Ki-67 activities in mitosis (Booth et al., 2014; Cuylen et al., 2016).

The cohesion between chromosomes during NR also requires the protein Barrier-to-Autointegration Factor (BAF) (Samwer et al., 2017; Sears and Roux, 2020). In interphase, BAF is partly restricted to the nuclear lamina where it interacts with DNA, histones, Lamin A/C and with transmembrane proteins of the INM that contain LEM (Lem2-Emerin-Man1) Domains (Barton et al., 2015; Sears and Roux, 2020). When the cell enters mitosis, BAF phosphorylation in its N-terminus (Thr2, Thr3, and Ser4) by vaccinia-related kinases (VRKs) induces its dissociation from DNA (Nichols et al., 2006; Birendra et al., 2017). When the cell exits mitosis, dephosphorylation allows BAF to interact with DNA again. Because BAF is a dimer, it acts as a cross-linker within and between chromosomes in late anaphase and telophase. The resulting tight clustering of the chromosomes excludes ER membranes, thereby preventing micronucleation during NER (Samwer et al., 2017).

BAF dephosphorylation in mitosis appears to depend on more than one phosphatase. In *C. elegans* and human cells, BAF recruitment to the DNA requires Ankle2/Lem4 (LEM4-L in *C. elegans*), a PP2A-interacting protein that localizes to the ER (Asencio et al., 2012; Snyers et al., 2018). PP2A can dephosphorylate BAF *in vitro* and it promotes BAF recruitment to chromosomes during NER. As human Ankle2 contains a LEM Domain that interacts with BAF, Ankle2 may serve as a bridging adaptor that facilitates BAF dephosphorylation by PP2A (Asencio et al., 2012). It was also shown that Ankle2 or LEM4-L can bind *C. elegans* VRK-1 and inhibit its phosphorylation of BAF (Asencio et al., 2012). Therefore, Ankle2 appears to promote the dephosphorylation of BAF by a dual mechanism simultaneously blocking its kinase and aiding its phosphatase. In *Drosophila*, BAF dephosphorylation and recruitment during NER partly depend on PP2A-Tws/B55 (Mehsen et al., 2018). Whether PP2A-B55 can also play this role in humans is unknown. However, it is known that Protein Phosphatase 4 (PP4) can dephosphorylate BAF at Ser4 and is required for BAF recruitment during NER in human cells (Zhuang et al., 2014). Interestingly, PP4 was also shown to regulate BAF in *Drosophila*, but earlier in mitosis. PP4 is targeted to centromeres through its regulatory subunit Falafel/PP4R3, which interacts with the centromeric protein CENP-C (Lipinszki et al., 2015). At this site, PP4 dephosphorylates a pool of BAF that is maintained at centromeres and promotes accurate chromosome segregation (Torras-Llort et al., 2020). Intriguingly, disrupting PP4-dependent maintenance of centromeric BAF in mitosis interferes with PP2A-dependent dephosphorylation of BAF during NR and results in NE defects (Torras-Llort et al., 2020). The relative contributions of PP2A-Ankle2, PP2A-B55 and PP4 to BAF function remain to be clarified. This problem is complexified by the presence of the three very close phosphorylation sites in the N-terminus of BAF which could differentially modulate its interactions with DNA and protein partners.

### Joining membranes and chromatin

**FIGURE 4:**
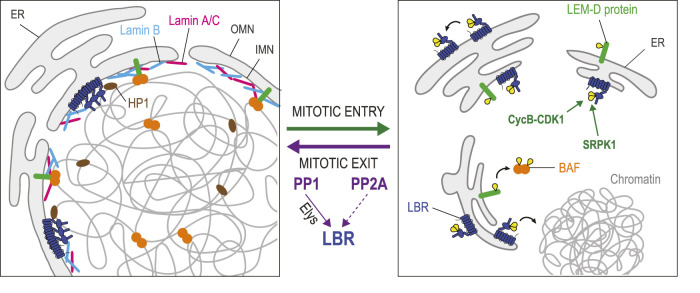
Dephosphorylation in joining nuclear membranes to chromatin during nuclear reassembly. In interphase, several integral proteins of the INM are in contact with chromatin-bound proteins. LEM-Domain proteins (green) interact with BAF (orange). Lamin B Receptor (LBR, dark blue) interacts with DNA, Lamin B (pale blue) and chromatin proteins including Heterochromatin Protein 1 (HP1, brown). LBR also multimerizes. During mitotic entry, phosphorylation of some LEM-Domain proteins promotes their dissociation from BAF. Phosphorylation of LBR by Cyclin B-CDK1 induces its dissociation from chromatin, while LBR phosphorylation by SPRK1 promotes the dissociation of LBR multimers. During mitotic exit, PP1 (assisted by Elys), and probably also PP2A, dephosphorylate LBR to promote the restoration of these interactions that contribute to nuclear reassembly. The dephosphorylation of some LEM-Domain proteins is probably required for their interactions with BAF, although the responsible phosphatase(s) is/are unknown. Yellow: phosphate groups.

Upon NEBD, nuclear membranes retract into the ER (Ungricht and Kutay, 2017). During mitotic exit, membranes are recruited back to chromatin from the ER to enable NER. Lamin B Receptor (LBR) is an integral protein of the INM that plays a key role in this process (Nikolakaki et al., 2017) (Figure 4). Its recruitment to chromatin depends on an interaction with the nucleocytoplasmic transport protein Importin *β* (Ma et al., 2007). During mitosis, Importin *β*, under regulation by RanGTP and chromatin-bound Rcc1, functions to release cargo proteins around chromosomes, including spindle assembly factors (Forbes et al., 2015). Similarly, Importin *β* also regulates NER around chromosomes at the end of mitosis.

The eight transmembrane domains of LBR contain a sterol reductase activity implicated in cholesterol synthesis (Olins et al., 2010). Its nucleoplasmic N-terminal domain interacts with heterochromatin through contacts with DNA and with several proteins including histones, Lamin B and Heterochromatin Protein 1 (HP1) (Ye and Worman, 1996; Ye et al., 1997; Nikolakaki et al., 2017). The N-terminal domain also contains multiple repeats of an arginine/serine (RS) motif that promote LBR multimerization in the INM (Nikolakaki et al., 1996; Nikolakaki et al., 2008). When cells enter mitosis, LBR is phosphorylated on RS motifs by the SRPK1 kinase, and at an adjacent site (Ser71) by CDK1 (Courvalin et al., 1992; Papoutsopoulou et al., 1999; Takano et al., 2004). These modifications cause the dissociation of LBR multimers and a resulting increase in mobility within the NE-ER network that promotes NEBD (Nikolakaki et al., 2008). Phosphorylation at Ser71 also suppresses LBR binding to chromatin *in vitro* (Takano et al., 2004). Conversely, dephosphorylation of LBR in its N-terminal domain at the end of mitosis is thought to promote its multimerization and interaction with chromatin. However, phosphorylation of LBR at Ser71 is required for its transient interaction with Importin *β* and its recruitment to nascent nuclei (Lu et al., 2010). Thus, the sequential phosphorylation and dephosphorylation of LBR appears to be required for its role in NER. The recruitment of LBR-containing mitotic membranes to chromatin is stimulated by PP1 in *Xenopus* egg extracts (Ito et al., 2007). In HeLa cells, LBR dephosphorylation by PP1 and/or PP2A determines the timing of ER membrane recruitment to chromosomes in telophase (Tseng and Chen, 2011). The mechanisms and functional importance of LBR dephosphorylation at different sites in the NER process are unclear.

Interactions between membranes and chromatin during NER are also aided by BAF. Once recruited on segregated chromosomes, BAF binds LEM-Domain proteins and Lamin A/C (Sears and Roux, 2020) (Figure 4). Phosphorylation of BAF on Thr3 and Ser4 strongly decreases BAF affinity for DNA, but it does not impair its binding to Lamin A/C or to the LEM-Domain protein Emerin *in vitro* (Marcelot et al., 2021). However, phosphorylation of Emerin and Man1 in mitosis disrupts their binding to BAF dimers in *Xenopus* extracts (Hirano et al., 2005; Hirano et al., 2009). The disruption of these complexes during mitosis may be necessary to allow the initial recruitment of free BAF on the telophase chromosomes prior to their interactions with LEM-Domain proteins. Which phosphatase(s) mediate(s) the dephosphorylation LEM-Domain proteins to license their binding to BAF during NER remains unknown. During interphase, BAF helps retain transmembrane LEM-Domain proteins at the INM as these can otherwise become partially dispersed in the ER membranes connected to the NE (Haraguchi et al., 2008). LEM-Domain proteins also engage in interactions with themselves, with lamins and with various proteins to regulate transcription on chromatin proximal to the lamina (Barton et al., 2015; Mirza et al., 2021). How these interactions are regulated by post-translational modifications in the cell cycle is not well understood.

### Reassembling the nuclear lamina

**FIGURE 5:**
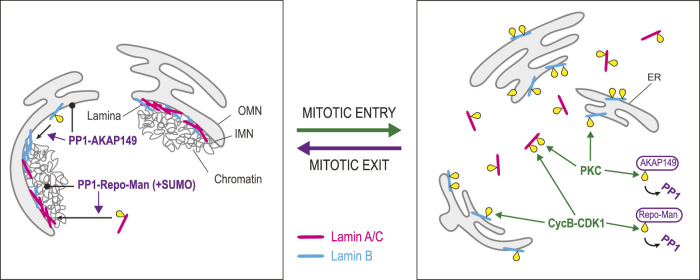
Dephosphorylation in lamina reassembly. Lamina disassembly during mitotic entry is triggered by phosphorylation of Lamins A/C (pink) and B (blue) by Cyclin B-CDK1 and PKC. Whiles Lamin A/C becomes scattered in the cytoplasm, Lamin B remains associated with ER membranes. At the same time, Cyclin B-CDK1 phosphorylates Repo-Man, preventing its interaction with PP1. Similarly, PKC phosphorylates AKAP149, preventing its interaction with PP1. Lamina reassembly during mitotic exit depends on the dephosphorylation of Lamins by PP1. The dephosphorylation of Lamin A/C depends on Repo-Man, which targets PP1 to chromatin. Moreover, SUMOylation of Repo-Man allows an interaction with Lamin A that facilitates its dephosphorylation by PP1. The dephosphorylation of Lamin B depends on AKAP149, a PP1 regulatory subunit that localizes to ER membranes. Yellow: phosphate groups.

In animal cells, the INM is associated with a protein network known as the lamina, which contacts chromatin and NPCs. This structure is disassembled during mitotic entry and reassembled during mitotic exit (Figure 5). The lamina is made of two types, each comprising multiple isoforms. B-Type Lamins (collectively referred to as Lamin B) are encoded by the *LMNB1* and *LMNB2* genes, while A/C-Type Lamins (collectively referred to as Lamin A/C) are encoded by the *LMNA* gene (de Leeuw et al., 2018; Liu and Ikegami, 2020). All Lamins contain an N-terminal head domain, a central rod domain and a C-terminal tail domain. Lamins form parallel dimers via their rod domain, and dimers assemble head-to-tail to form polymers that further assemble into anti-parallel filaments. As cells enter mitosis, CDK1 and PKC induce the disassembly of the lamina by phosphorylating Lamins A/C and B (Heald and McKeon, 1990; Peter et al., 1990; Peter et al., 1991; Eggert et al., 1993; Goss et al., 1994; Georgatos et al., 1997; Kuga et al., 2010; Liu and Ikegami, 2020). Phosphorylated Lamin A/C becomes dispersed in the cytoplasm in mitosis while phosphorylated Lamin B remains associated with membranes (Georgatos et al., 1997). This difference is attributed to the fact that Lamin B is anchored to membranes through a C-terminal farnesylated cysteine residue, while Lamin A/C lacks this modification (Farnsworth et al., 1989; Liu and Ikegami, 2020).

Lamina reassembly largely depends on PP1 enzymes. Biochemical work pointed at a prominent role for PP1 in the dephosphorylation of mitotic sites on Lamin B (Thompson et al., 1997). It was later shown that the PP1-interacting protein AKAP149 is required for this process in the cell (Steen et al., 2000). As AKAP149 is an integral protein of the ER, it is well positioned to bring PP1 activity around chromosomes in telophase when the NE reassembles from the ER. Interestingly, the phosphorylation of AKAP149 by PKC disrupts its interaction with PP1, suggesting that PKC may prevent PP1-dependent Lamin B dephosphorylation in early mitosis (Kuntziger et al., 2006). Dephosphorylation of Lamin A requires Repo-Man, an alternative PP1-interacting protein that targets PP1γ to chromosomes in telophase (Trinkle-Mulcahy et al., 2006; Moriuchi and Hirose, 2021; Huguet et al., 2022). SUMOylation of Repo-Man allows its binding to a SUMO-interacting motif in Lamin A and the dephosphorylation of Lamin A at Ser22, promoting lamina reassembly (Moriuchi and Hirose, 2021; Huguet et al., 2022). During mitotic entry, CDK phosphorylation of Repo-Man prevents its binding to PP1, potentially preventing Lamin A dephosphorylation (Vagnarelli et al., 2011; Qian et al., 2015). Curiously, AKAP149 and Repo-Man do not appear to be widely conserved among animals. For example, *Drosophila* or *C. elegans* do not have predicted orthologs of these proteins, suggesting that alternative mechanisms can mediate Lamins dephosphorylation by PP1 or another phosphatase.

PP2A may also contribute to Lamins dephosphorylation at the end of mitosis. The phosphorylation levels of several sites on Lamins A and B are sensitive to the expression levels of cellular PP2A inhibitors (Kauko et al., 2020). However, whether these dependencies reflect a role for PP2A in promoting lamina reassembly after mitosis remains unexplored.

### Nuclear pore complex reassembly

**FIGURE 6:**
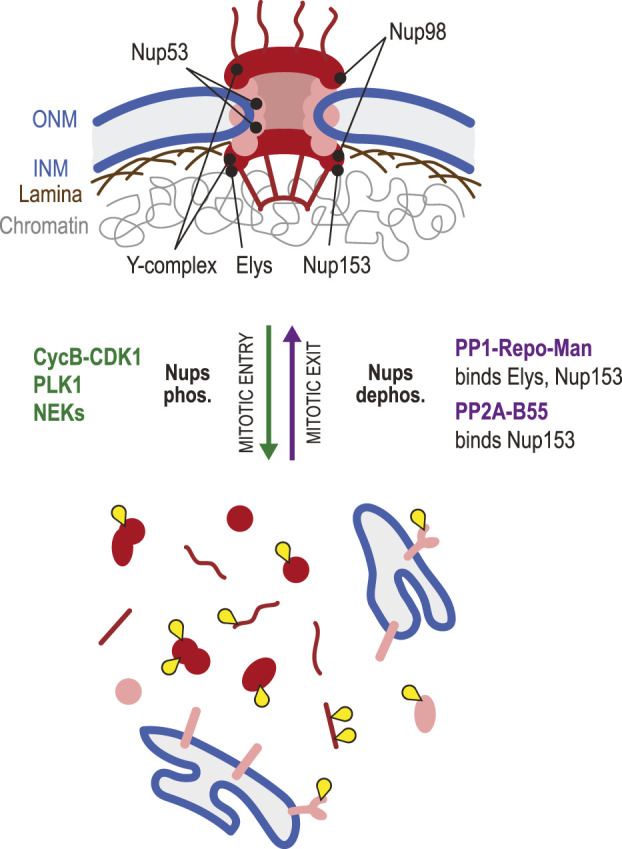
Dephosphorylation in nuclear pore complex reassembly. NPCs (red/pink) are large assemblies of Nups that control nucleocytoplasmic exchanges. During mitotic entry, NPCs are disassembled due to the combined action of Cyclin B-CDK1, PLK1 and NEKs that phosphorylate multiple Nups, disrupting their interactions. In this process, phosphorylation of Nup98 allows an initial increase in permeability of the pores. Subsequent phosphorylation of Nup53 is key to the dismantling of NPCs. In mitosis, some Nups are scattered in the cytoplasm as monomers or subcomplexes, while transmembrane Nups remain associated with ER membranes. During mitotic exit, Elys and Nup153 are recruited on chromatin. Both proteins bind PP1-Repo-Man, which probably promotes the local dephosphorylation of Nups required for the incorporation in reassembling NPCs. Elys interacts with, and is required for the recruitment of the Y-complex (Nup107-Nup160). PP2A-B55 interacts with Nup153, promotes its dephosphorylation and is required for its recruitment. Yellow: phosphate groups.

Molecular exchanges between the nucleus and the cytoplasm are controlled by NPCs (Wente and Rout, 2010). These are large assemblies of some 30 different nucleoporin proteins (Nups) each present in multiple copies to form rings with an 8-fold symmetry that define holes in the NE (Fernandez-Martinez and Rout, 2021). NPCs are disassembled during mitotic entry and reassembled during mitotic exit (Figure 6). At the early stage of NEBD, CDK1 cooperates with PLK1 and NIMA-related kinase (NEKs) to phosphorylate multiple nucleoporins, contributing to the efficient disassembly of NPCs (Laurell et al., 2011; de Castro et al., 2017b; Linder et al., 2017; Martino et al., 2017; Kutay et al., 2021). Initiation of NPC disintegration requires hyperphosphorylation of the gatekeeper Nup98 and the scaffold nucleoporin Nup53. Hyperphosphorylation of Nup98 at its C-terminal domain disrupts its interactions with other Nups around the ring scaffold, leading to an increase in NE permeability and promoting NPC disassembly (Laurell et al., 2011; Linder et al., 2017). Similarly, multiple phosphorylations of Nup53 by CDK1 and PLK1 result in its complete dissociation from the nuclear pore inner ring complex and the pore membrane (Linder et al., 2017; Martino et al., 2017). The phosphorylation of many other Nups promotes NPC disassembly, and Nups exist as monomers or subcomplexes, in soluble or membrane-associated forms during mitosis (Kutay et al., 2021).

How dephosphorylation of Nups promotes NPC reassembly in the NE at the end of mitosis is still poorly understood. A role for PP1 or PP2A in this process was suggested by the observation that injection of okadaic acid (a PP1 and PP2A inhibitor) in *Drosophila* embryos prevents NPC reassembly (Onischenko et al., 2005). A phosphoproteomic study of early mitotic exit in human cells found that Nup107, Pom121C, and Nup53 were dephosphorylated first, followed by Nup93 and Nup188, which correlates with the specific order of recruitment of these proteins to the reforming NE (McCloy et al., 2015). A search for PP2A-B55 targets identified the Nups Nup153, Nup107 and NDC1 as probable substrates during mitotic exit, and the ability of PP2A-B55 to bind Nup153 is required for Nup153 recruitment to the NE during this transition (Cundell et al., 2016). Depletion of B55 also delays the recruitment of other Nups examined, reinforcing the idea that PP2A-B55 plays an essential role in timely NPC reassembly, possibly by dephosphorylating multiple Nups (Cundell et al., 2016). Nup153 is a chromatin-associated protein that is targeted to chromosomes in late anaphase (Bodoor et al., 1999). This Nup can interact directly with PP1 through a PP1-docking motif (Moorhead et al., 2008). It also forms a complex with PP1 by binding its partner Repo-Man (Vagnarelli et al., 2011; de Castro et al., 2017a). Nup153 recruits PP1-Repo-Man on chromosomes in late anaphase and, intriguingly, Repo-Man is also required for Nup153 recruitment in a PP1-independent manner (Vagnarelli et al., 2011; de Castro et al., 2017a). Thus, Nup153 appears to serve as a pivotal point of regulation in dephosphorylation-dependent NPC reassembly in mitotic exit.

Elys is another chromatin-associated Nup that plays key roles in post-mitotic NPC assembly (Rasala et al., 2006; Shevelyov, 2020). As chromosomes begin to decondense in telophase, Elys is recruited on DNA (Rasala et al., 2008). In turn, Elys recruits the Y-complex (Nup107-160 subcomplex) of the NPC in late anaphase (Franz et al., 2007). In *C. elegans*, Mel-28/Elys binds PP1 on decondensing chromatin, and this pool of PP1 appears to promote NR in several ways (Hattersley et al., 2016). In human cells, Elys was shown to promote PP1-dependent dephosphorylation of LBR and its recruitment to the reassembling NE (Clever et al., 2012; Mimura et al., 2016). Thus, Elys may play a conserved double role in NPC assembly, by directly recruiting Nups and by promoting the PP1-dependent dephosphorylation and recruitment of other NE proteins. Although multiple phosphorylation sites have been detected on Elys (Phosphosite.org) (Hornbeck et al., 2015), the roles of its phosphoregulation remain unexplored.

### Nuclear envelope sealing

NER culminates with the sealing of holes and gaps in membranes added from the mitotic ER. This sealing is required to restore the permeability barrier that completely relies on NPCs for nucleocytoplasmic exchanges. This event is mediated by the ESCRT-III machinery, a set of proteins that form filaments assisted by the VPS4 ATPase to shape the membranes (Olmos et al., 2015; Vietri et al., 2015). ESCRT-III recruitment depends on the binding of its CHMP7 subunit to LEM2 (Gu et al., 2017). Through its LEM domain, LEM2 is recruited by BAF on telophase chromosomes (von Appen et al., 2020). There, LEM2 oligomerizes into a phase-separated structure that interacts with MTs and promotes ESCRT-III function. These proteins are inhibited by reversible phosphorylation in their NER functions in at least two ways. First, mitotic phosphorylation of LEM2 in a disordered region C-terminal to its LEM domain, disrupts its ability to oligomerize and interact with MTs (von Appen et al., 2020). Second, CHMP7 phosphorylation at two CDK sites downregulates its ability to interact with LEM2 (Gatta et al., 2021). The identity of the putative phosphatase(s) involved in counteracting these phosphorylation events to promote NER is unknown, but LEM2 has been found to interact with PP1, which is therefore a candidate (Asencio et al., 2012).

### Chromosome decondensation

**FIGURE 7:**
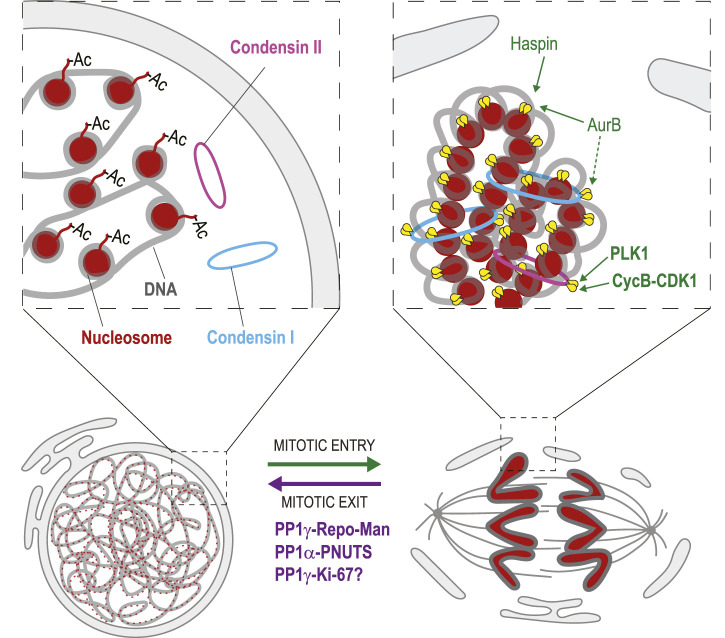
Dephosphorylation in chromosome decondensation. During mitotic entry, phosphorylation of Histone H3 by Haspin and Aurora B promotes interactions between nucleosomes (red) that contribute to chromosome condensation. Phosphorylation of Condensin II (pink) by PLK1 and CDK1 is required for its loading on mitotic chromosomes. Aurora B activity is required for the loading of Condensin I (blue). During mitotic exit, Histone H3 is dephosphorylated, which is mediated at least in part by PP1γ-Repo-Man. This event is required for the acetylation (Ac) of nucleosomes on Histone H4 tails that disrupts interactions between nucleosomes. Condensin dephosphorylation presumably contributes to their dissociation from chromosomes. PP1α-PNUTS promotes chromosome decondensation although its precise targets are unknown. PP1γ-Ki-67 may also contribute to chromosome decondensation. Yellow: phosphate groups. This conceptual cartoon is not intended to display structural organization in precise detail.

The sorting and segregation of chromatids in mitosis require chromosome condensation and decatenation during mitotic entry (Batty and Gerlich, 2019). During mitotic exit, chromosomes have to decondense to allow the shaping of interphase nuclei where transcription and other nuclear processes can occur normally (Figure 7). This cycle is not completely understood in physical, topological or biochemical terms. Nevertheless, several reversible phosphorylation events have been shown to play roles. Chromosome condensation involves at least the induction of interactions between nucleosomes and the looping of chromatin by Condensin complexes (Antonin and Neumann, 2016; Kagami and Yoshida, 2016).

In interphase, acetylation of Histone 4 at Lys16 prevents interactions between nucleosomes (Shogren-Knaak et al., 2006). As cells prepare for mitosis, phosphorylation of Histone 3 at Thr3 by Haspin kinase allows Aurora B to phosphorylate Histone 3 at Ser10 (Hendzel et al., 1997; Kelly et al., 2010; Wang et al., 2010). In turn, Ser10 phosphorylation allows the deacetylation of Histone 4 at Lys16 by Hst2, permitting interactions between nucleosomes that promote chromosome condensation (Wilkins et al., 2014).

Mitotic chromosome condensation also involves multiple phosphorylation events of Condensin complexes. In animals, there are two Condensin complexes (I and II), each composed of 5 subunits and required in the condensation process (Kalitsis et al., 2017). Condensin II is recruited first on prophase chromosomes, while Condensin I comes after NEBD and is more dynamic (Gerlich et al., 2006). The initiation of condensation requires Condensin II phosphorylation by CDK1 and PLK1 (Abe et al., 2011; Kagami et al., 2017). Aurora B activity is needed for the recruitment of the Condensin I complex to mitotic chromosomes in yeast, flies, worms and vertebrates (Giet and Glover, 2001; Kaitna et al., 2002; Lipp et al., 2007; Takemoto et al., 2007; Nakazawa et al., 2011; Tada et al., 2011). In fission yeast, where only one Condensin complex exists, phosphorylation of the Cnd2 Kleisin subunit by Aurora B is required for Condensin recruitment to mitotic chromosomes, which reaches its maximum in anaphase (Nakazawa et al., 2011; Tada et al., 2011). Similarly, in human cells, phosphorylation of CAP-H, the kleisin subunit of Condensin I, is required for the correct localization of the complex to mitotic chromosomes through CAP-H interaction with H2A and H2A.Z histones (Tada et al., 2011). Phosphorylation of CAP-G by CDK1 is also required for Condensin I localization to mitotic chromosomes (Murphy and Sarge, 2008). In budding yeast, Condensin is phosphorylated by Cdc5 (Polo kinase), which stimulates its condensing activity in anaphase (St-Pierre et al., 2009).

The decondensation of chromosomes that occurs when nuclei reassemble during mitotic exit requires dephosphorylation events. Reversal of Histone 3 phosphorylation in its N-terminal tail is presumably needed to allow its reacetylation and a consequent loss of inter-nucleosomal interactions required for chromosome decondensation. At the end of mitosis, PP1γ dephosphorylates Histone 3 at Thr3, Ser10 and other sites, a function that requires the PP1γ targeting protein Repo-Man (Qian et al., 2011). However, the loss of Repo-Man does not result in obvious chromosome decondensation defects (Vagnarelli et al., 2011). This suggests that other events must occur to enable decondensation, likely including Condensin dephosphorylation. However, surprisingly little is known about the functional importance and mechanisms of Condensin complexes dephosphorylation for chromosome decondensation, nor do we know precisely which phosphatase(s) are responsible for each event.

PP1γ is also targeted to late mitotic chromosomes by Ki-67 (Booth et al., 2014), and this complex may promote chromosome decondensation. In addition, chromosome decondensation is stimulated by PP1α and its partner PNUTS, which recruits PP1α to reassembling nuclei (Landsverk et al., 2005; Lee et al., 2010). Although it is tempting to suggest that PP1γ-Ki-67 and PP1α-PNUTS dephosphorylate Histones and Condensins, the precise targets of these enzymatic complexes are still unknown.

PP2A plays a specialized role in regulating Condensin I activity in mitosis. The TATA-Binding Protein interacts and retains PP2A on chromatin to keep Condensin I dephosphorylated, preventing complete condensation at sites that are transcriptionally active in interphase (Xing et al., 2008). This mechanism provides a gene activity memory through mitosis. PP6 was recently found to oppose CK2 phosphorylation on Condensin I, and to regulate chromosome condensation in mitosis (Rusin et al., 2015). However, the proposed regulation plays a role before mitotic exit in this case. In fission yeast, it was shown that an RNA processing complex, in association with the PP1 and Ssu72 phosphatases, opposes Condensin-dependent chromosome condensation (Vanoosthuyse et al., 2014). These studies constitute entry points into the complex and still open problem of Condensin regulation by dephosphorylation.

Dephosphorylation during mitotic exit could also promote chromosome decondensation indirectly. One mechanism may involve NuMa, an abundant protein that regulates the spindle in mitosis and localizes to the nucleus in interphase, where it apparently contributes to the establishment of a nuclear matrix (Radulescu and Cleveland, 2010). NuMa interacts with chromatin in interphase, and dissociates from it upon mitotic entry, possibly due to its phosphorylation by Cyclin B-CDK1 (Rajeevan et al., 2020). Truncation of the C-terminal region of NuMa that binds chromatin results in chromosome decondensation defects during mitotic exit (Rajeevan et al., 2020). In addition, inactivation of kinases specifically in the reassembling nucleus can promote the dephosphorylation of their targets. For example, the Cdc48/p97 ATPase mediates the removal of Aurora B from chromatin during mitotic exit (Ramadan et al., 2007). Disrupting this process leads to decondensation defects.

### Chromatin and nucleolus organization

In interphase, chromatin is highly organized in the nucleus. Several regions of heterochromatin are tethered to the lamina at the NE, forming lamina-associated domains (LADs) where transcription tends to be silenced (van Steensel and Belmont, 2017). HP1 protein contributes to this topology, by binding Histone H3 trimethylated at Lys9 on heterochromatin and associating with LBR (Ye and Worman, 1996; Ye et al., 1997; Bannister et al., 2001; Jacobs et al., 2001; Lachner et al., 2001). NE-associated proteins including Lamins, LEM-Domain proteins and NPCs contribute to transcriptional regulation (Mirza et al., 2021; Nazer, 2022; Sumner and Brickner, 2022). In mitosis, this topological organization is largely lost. Phosphorylation of Histone H3 at Ser10 by Aurora B prevents HP1 binding to Histone H3 (Fischle et al., 2005; Hirota et al., 2005). When the NE reassembles at the end of mitosis, the links between some heterochromatin and the NE are restored, ensuring that the appropriate heterochromatin regions regain their peripheral positions in the nucleus. This is in part due to the dephosphorylation of Histone H3 Ser10 by Repo-Man-associated PP1, which allows Histone H3 to bind HP1 (Vagnarelli et al., 2011). This event is facilitated by the recruitment of Repo-Man to Nup153 (Vagnarelli et al., 2011; de Castro et al., 2017a). Work in fruit flies suggests that timely recruitment of HP1 on heterochromatin may also promote the assembly of the NE (Warecki and Sullivan, 2018).

The nucleolus is a large intranuclear structure where ribosomal DNA is transcribed, ribosomal RNA matures and ribosomal subunits are assembled (Boisvert et al., 2007). Some cell types contain several nucleoli. The nucleolus is disassembled in mitosis. This dissolution requires a high level of Cyclin B-CDK1 activity (Gavet and Pines, 2010). One of its phosphorylation substrates is nucleophosmin/B23, which functions in ribosomal RNA processing in the nucleolus (Okuwaki et al., 2002). Phosphorylation of nucleophosmin disrupts its RNA-binding activity and its nucleolar localization in prophase (Okuwaki et al., 2002; Negi and Olson, 2006). Nucleophosmin is a largely disordered protein and its integration into the nucleolus requires LLPS (Mitrea et al., 2016). It was recently shown that multisite phosphorylation of nucleophosmin in its disordered region disrupts its ability to undergo LLPS, promoting its dissociation from the nucleolus (Yamazaki et al., 2022). This occurs because phosphorylation of nucleophosmin disrupts charge blocks in its amino-acid sequence that can promote LLPS. Interestingly, the same study identified several other nucleolar proteins with charged disordered regions that become hyperphosphorylated in mitosis, possibly disrupting their LLPS to favor nucleolar disassembly (Yamazaki et al., 2022). Cyclin B-CDK1 also phosphorylates proteins of the RNA Pol I machinery to terminate the transcription of ribosomal genes in mitosis (Hernandez-Verdun, 2011). Some of these phosphorylation events may also contribute to nucleolar disassembly.

The reassembly of the nucleolus presumably requires the dephosphorylation of several of its proteins. A phosphomimetic mutant of nucleophosmin is unable to undergo LLPS and thus fails to reintegrate the nucleolus after mitosis. (Yamazaki et al., 2022). The dephosphorylation of multiple nucleolar proteins could similarly contribute to restoring their interactions with proteins and nucleic acids within the nucleolus, and in this way, contribute to the reassembly of this membraneless organelle. The dephosphorylation of Ki-67 may also promote its recruitment to the nucleolus.

Nucleolar reassembly begins in telophase and progresses until G1 when rDNA transcription and ribosomal assembly become fully operational and coordinated (Hernandez-Verdun, 2011). In the nascent nucleus, transient prenucleolar bodies (PNBs) assemble, where nucleolar processing complex assemble before they later reach the nucleolus (Savino et al., 2001). PP1γ and PP1β are present in PNBs and in the nucleolus, where they are responsible for most of the phosphatase activity (Trinkle-Mulcahy et al., 2003; Lesage et al., 2005). Thus, these PP1 isoforms are possibly responsible for the dephosphorylation of nucleophosmin and other Cyclin B-CDK1 substrates among nucleolar proteins. The protein RRP1B recruits PP1γ and PP1β to the nucleolus and it was suggested that these complexes may be involved in nucleolar reassembly after mitosis (Chamousset et al., 2010). In addition, depletion of the PP1-interacting nucleolar protein Ki-67 results in smaller and less numerous nucleoli (Booth et al., 2014). The same study showed that Ki-67 is found in complex with nucleophosmin and promotes its dephosphorylation. Whether the ability of Ki-67 to interact with PP1 is required for these functions in nucleolar reassembly was not examined. We suggest that Ki-67 and RRP1B may collaborate in this process.

### Kinetochore disassembly

**FIGURE 8:**
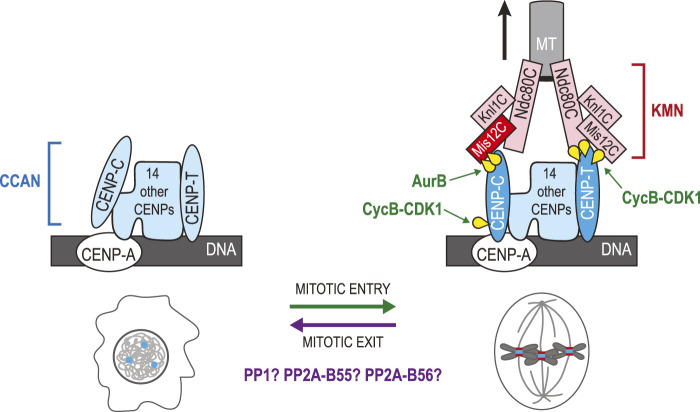
Phosphoregulation of kinetochore assembly and disassembly in the mitotic cycle. CCAN proteins (blue) are assembled at centromeres defined by the presence CENP-A in nucleosomes. During mitotic entry, phosphorylation events promote the recruitment of KMN proteins (red/pink) onto the CCAN. Phosphorylation of Dsn1 in the Mis12C by Aurora B promotes binding of the complex to CENP-C. Phosphorylation of CENP-C by Cyclin B-CDK1 promotes its interaction with CENP-A. In parallel, phosphorylation of CENP-T by Cyclin B-CDK1 promotes its interactions with the Ndc80C and with the Mis12C. The Ndc80C binds MTs of the mitotic spindle until chromosomes have segregated. During mitotic exit, the KMN is disassembled from KTs as interphase nuclei assemble. Dephosphorylation of Dsn1, CENP-C and CENP-T is presumably required for this transition. PP1, PP2A-B55 and PP2A-B56 are probably involved in this regulation. The schematic representation of the KT is based on a previous review (Hara and Fukagawa, 2020). Yellow: phosphate groups.

The segregation of chromosomes on the mitotic spindle requires KTs, a large assembly of several protein complexes that control the attachment and dynamics of chromosomes at the level of their centromeres (Musacchio and Desai, 2017). Since the assembly of mitotic KTs requires phosphorylation of some of their subunits, dephosphorylation events are thought to be required for KT disassembly (Figure 8) (Hara and Fukagawa, 2018). Before discussing how KTs are disassembled, it is essential to briefly introduce how they are assembled at the beginning of mitosis. Chromosomes contain the Constitutive Centromere Associate Network (CCAN), a protein complex that resides on centromeric chromatin throughout the cell cycle (Hara and Fukagawa, 2017). The CCAN interacts with nucleosomes containing CENP-A, a Histone H3 variant that is specific to centromeres (McKinley and Cheeseman, 2016). The CCAN also serves as a platform for the assembly of the KNL1, Mis12 and Ndc80 complexes (KMN) network in mitosis (Musacchio and Desai, 2017; Hara and Fukagawa, 2018). The Ndc80 complex of the KMN interacts with MTs (Cheeseman et al., 2006; Wei et al., 2007). Additional accessory proteins are recruited to KTs and provide other contacts with MTs (Monda and Cheeseman, 2018). Another important function of KTs is to recruit components of the Spindle Assembly Checkpoint (SAC) that ensures that chromosomes are attached and under bipolar tension before anaphase is triggered (Musacchio, 2015).

Mitotic kinases promote KT assembly by phosphorylating some of their subunits as cells enter mitosis (Figure 8). In vertebrates, two proteins of the CCAN directly bind and recruit KMN network complexes: CENP-C and CENP-T (Hori et al., 2008; Gascoigne et al., 2011). The Mis12 complex binds to CENP-C when Aurora B phosphorylates its Dsn1 subunit at Ser100 and Ser109 in its basic tail (Yang et al., 2008; Welburn et al., 2010; Kim and Yu, 2015; Rago et al., 2015). Structural studies have shown that when unphosphorylated at these sites, this basic tail of Dsn1 is sequestered by binding to the Mis12 subunit (Petrovic et al., 2016). In addition, phosphorylation of CENP-C at Thr734 by Cyclin B-CDK1 promotes its interaction with CENP-A within the CCAN (Watanabe et al., 2019). In parallel to these events, human CENP-T is phosphorylated at two CDK1 sites, Thr11 and Thr85, as cells enter mitosis (Rago et al., 2015). These modifications in CENP-T expose two binding sites for the Spc24-Spc25 dimers of the Ndc80 complex (Huis In 't Veld et al., 2016; Nishino et al., 2013). In addition, phosphorylation of CENP-T at Ser201, a non-canonical site CDK1 site, promotes the binding of the Mis12 complex, bringing with it an additional Ndc80 complex to the KT (Huis In 't Veld et al., 2016; Nishino et al., 2013). There are potentially other phosphorylation events that contribute to reinforcing the assembly of mitotic KTs or their ability to interact with MTs.

It seems reasonable to suppose that Dsn1, CENP-C, and CENP-T dephosphorylation may promote the dissociation of the KMN from the CCAN, prompting the disassembly of KTs during mitotic exit (Figure 8). PP1 was shown to counteract Aurora B-dependent KT assembly in humans, frogs and budding yeast (Emanuele et al., 2008). Moreover, depletion of two PP1 regulator subunits, Sds22 and Repo-Man caused hyperphosphorylation of Dsn1 at Ser100 on mitotic chromosomes (Wurzenberger et al., 2012). Depletion of these PP1 subunits caused chromosome segregation defects; however potential defects in KT disassembly in telophase were not examined. When Aurora B, as part of the Chromosomal Passenger Complex (CPC), re-localizes to the central spindle, the Aurora B/PP1 balance at KTs likely flips towards PP1, promoting Dsn1 dissociation from CENP-T. As CENP-T is phosphorylated by CDK1, it has been proposed that PP2A-B55 may be responsible for its dephosphorylation as this phosphatase efficiently targets CDK phospho-motifs (Hara and Fukagawa, 2018). A similar scenario can be proposed for the dephosphorylation of the CDK1 site on CENP-C. However, PP2A-B55 does not concentrate on KTs. PP2A-B56 and PP1 may be better poised to fill this function as they are targeted to KTs during mitosis (Garvanska and Nilsson, 2020). In *C. elegans*, a pool of MEL-28/ELYS at KTs recruits PP1, and this function is required for KT disassembly in meiosis I (Hattersley et al., 2016). Future studies should determine the precise contributions of each dephosphorylation event in the return of centromeres to their interphase state, and which phosphatases predominantly regulate which events. More generally, it would be interesting to learn the cellular consequences of a failure to disassemble mitotic KTs as interphase nuclei reassemble.

### Temporal regulation of dephosphorylation in nuclear reassembly

The correct and progressive transition from mitosis to interphase requires mechanisms that impose the order of events. Some of these mechanisms act on the activity or targeting of phosphatases (Figure 2B). PP2A-B55 is inhibited during mitotic entry through the Greatwall (Gwl) kinase (Yu et al., 2004; Castilho et al., 2009; Mochida et al., 2009; Vigneron et al., 2009). At this time, the Gwl/MASTL kinase is activated by phosphorylation of CDK sites (Yu et al., 2006; Blake-Hodek et al., 2012). In turn, Gwl phosphorylates Endosulfine proteins (ENSA and Arpp19 in human cells) that thereby become potent and selective competitive inhibitors of PP2A-B55 (Gharbi-Ayachi et al., 2010; Mochida et al., 2010). Endosulfines are substrates of PP2A-B55 that are dephosphorylated very inefficiently by the enzyme but that have a relatively high affinity for it, compared to its other cellular substrates (Williams et al., 2014). As a result, PP2A-B55 is kept inactive towards its other substrates as long as phosphorylated Endosulfines are present and regenerated by Gwl.

Mitotic exit begins when bipolar attachment of chromosomes satisfies the SAC, allowing activation of the Anaphase Promoting Complex/Cyclosome (APC/C) (Lara-Gonzalez et al., 2021). The APC/C ubiquitinates several proteins, including Securin and Cyclin B, thereby targeting them for degradation by the 26 S proteasome (Sivakumar and Gorbsky, 2015). Both Securin and Cyclin B-CDK1 inhibit Separase, the protease responsible for cleaving cohesins that keep sister chromatids cohesion until metaphase (Ciosk et al., 1998; Uhlmann et al., 1999; Uhlmann et al., 2000; Stemmann et al., 2001; Gorr et al., 2005). Thus, APC/C activation allows sister chromatids to segregate in anaphase (Figure 2B). After anaphase onset, the decreased Cyclin B levels cannot sustain Gwl activation by CDK1, and Gwl is inactivated by PP1 (Heim et al., 2015; Ma et al., 2016; Rogers et al., 2016; Ren et al., 2017). The time needed for these events and for PP2A-B55 to dephosphorylate Endosulfines defines a delay between anaphase and late mitotic events that depend on PP2A-B55. When Gwl or ENSA are inactivated experimentally, cytokinesis is attempted prematurely in HeLa cells exiting mitosis (Cundell et al., 2013). Similarly, mouse embryonic fibroblasts knocked-out for Arpp19 exhibit a premature recruitment of Nups on chromosomes in anaphase (Hached et al., 2019). Thus, the Gwl-Endosulfine module appears to act as a timer that determines the onset of PP2A-B55 activity towards NR. Another level of temporal control is provided by the phosphorylation of PP2A catalytic at Thr304 by Cyclin B-CDK1 during mitotic entry, which strongly reduces the incorporation of B55 into PP2A holoenzymes (Nasa et al., 2020). Dephosphorylation of PP2A catalytic presumably promotes PP2A-B55 functions in NR but the timing, mechanism and contribution of this event have not yet been characterized.

The targeting of PP1 to chromatin by Repo-Man is inhibited in early mitosis. CDK phosphorylation of Repo-Man prevents its loading on chromosomes until anaphase when mitotic cyclins are degraded (Vagnarelli et al., 2011; Qian et al., 2015). CDK phosphorylation of Repo-Man additionally prevents its binding to PP1 (Vagnarelli et al., 2011; Qian et al., 2015). Preventing the phosphorylation of Repo-Man by mitotic CDKs causes the premature recruitment of Repo-Man on prometaphase chromosome and the premature dephosphorylation of PP1 substrates (Qian et al., 2015). In parallel, PP1 is inhibited in early mitosis by direct phosphorylation at a CDK site and by its binding of Inhibitor-1 phosphorylated by PKA (Dohadwala et al., 1994; Wu et al., 2009). In addition, the phosphorylation of Repo-Man at a different site inhibits its binding to Importin β, and its dephosphorylation enables Importin β recruitment which promotes NER (Vagnarelli et al., 2011). Incidentally, PP1 is known or strongly suspected to be responsible for the dephosphorylation of: Repo-Man at CDK sites, PP1’s inhibitory CDK site, and Inhibitor-1 at its activating PKA site (Wu et al., 2009; Vagnarelli et al., 2011; Vagnarelli and Earnshaw, 2012; Qian et al., 2015). This situation likely allows a rapid and switch-like amplification of PP1 activity on anaphase chromosomes (Vagnarelli and Earnshaw, 2012). It is not yet clear how these events promote timely PP1 activation on chromosomes and how they may be coupled with timely PP2A reactivation to coordinate the events of NR.

An additional layer of temporal regulation is encoded directly in the primary sequences of the phosphatase substrates, with respect to the enzyme’s motif preference. Enzymes tend to process their cellular substrates in an order that reflects a combination of their decreasing order of affinity as well as their decreasing catalytic constants. PP2A-B55 favors phosphothreonine residues over phosphoserine residues (Hein et al., 2017; Touati et al., 2018). PP2A-B55 also favors substrates containing basic amino-acid residues near its dephosphorylation site, seemingly because of acidic residues on the B55 substrate binding site (Xu et al., 2008; Cundell et al., 2016; Touati et al., 2018; Holder et al., 2020). Thus, substrates whose target site contains a phosphothreonine and adjacent basic residues tend to be dephosphorylated first by PP2A-B55 during mitotic exit. This description generally applies to mitotic spindle substrates of PP2A-B55 (Cundell et al., 2016). Conversely, targets containing phosphoserine and no basic residues tend to be dephosphorylated last by PP2A-B55. Such proteins include NE proteins such as Nups whose dephosphorylation likely promotes NR after spindle disassembly (Cundell et al., 2016).

Although less thoroughly characterized, the dephosphorylation motif preference of PP1 appears to be more relaxed compared to that of PP2A-B55. Instead, PP1 relies more heavily on the recognition of Short Linear Motifs (SLiMs) in many of its regulatory subunits, docking proteins and substrates (Bollen et al., 2010; Heroes et al., 2013; Garvanska and Nilsson, 2020). The most common PP1 SLiM is the 1RVxF docking motif (Egloff et al., 1997). In the mechanisms described above, SLiMs facilitate PP1 interactions with at least Repo-Man, Ki-67, AKAP149 and PNUTS and probably also Elys and Nup153 (Landsverk et al., 2005; Kuntziger et al., 2006; Trinkle-Mulcahy et al., 2006; Moorhead et al., 2008; Booth et al., 2014; Nasa et al., 2018). In *S. pombe*, PP1 also binds PP2A-B55 and PP2A-B56 through SLiMs to promote their reactivation during mitotic exit (Grallert et al., 2015). This mechanism coupling three phosphatases is likely conserved in vertebrates where the SLiMs in B55 and B56 are conserved. PP2A-B56 and PP4 also use their regulatory subunits to recognize SLiMs in their targets (Hertz et al., 2016; Wang et al., 2016; Ueki et al., 2019; Garvanska and Nilsson, 2020; Karman et al., 2020). Thus, additional contributions of these enzymes to the NR process that may be identified in the future could involve their binding to SLiMs.

The global order of dephosphorylation events in the transition from mitosis to interphase, including NR, largely reflects the substrate preferences of both PP2A-B55 and PP1 which act in a coordinated manner (McCloy et al., 2015; Touati et al., 2018; Holder et al., 2020). It also reflects the phosphorylation kinetics of mitotic kinases’ substrates and the changes in these kinase activities as the cell cycle progresses (Bouchoux and Uhlmann, 2011).

### Spatial regulation of dephosphorylation in nuclear reassembly

**FIGURE 9:**
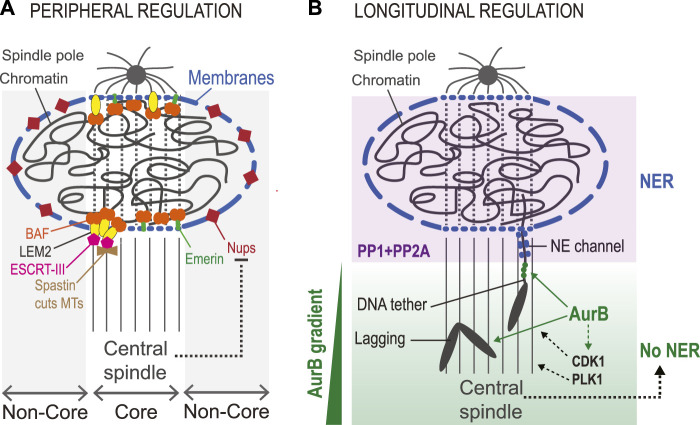
Elements of spatial regulation in nuclear reassembly. **(A)**. Peripheral regulation. The presence of the central spindle during NR defines the core region, where some proteins are preferentially recruited. These include BAF, LEM-Domain protein including LEM2 and Emerin, ESCRT-III (the membrane sealing enzyme) and Spastin (the MT-severing enzyme). The region not in contact with the central spindle is the non-core region, where other proteins of the NE are preferentially recruited, including Nups. The central spindle likely contributes to inhibiting the recruitment of non-core proteins associated with large ER sheets (long blue segments). Transmembrane proteins recruited to the core region tend to depend on BAF and may be recruited through smaller membrane structures (short blue segments) that can infiltrate the central spindle. **(B).** Longitudinal regulation. An Aurora B (AurB) gradient from the equator to the poles regulates NER. Lagging chromosomes in anaphase show delays in NER and in chromosome decondensation. In *Drosophila*, acentric chromosome fragments remain connected to the main chromosome mass by a DNA tether that defines a channel in the NE until the chromosome fragment has been integrated into the nucleus. Aurora B localizes to these DNA tethers (green dots). These delays presumably depend on the phosphorylation of Aurora B substrates, and possibly also on substrates of CDK1 and/or PLK1 whose localization and activity are promoted by Aurora **(B)**. When segregation is completed, these proteins are presumably dephosphorylated by PP1 and PP2A enzymes to promote NER. In addition, the central spindle likely contributes to delayed NER on lagging chromosomes.

Nuclear reassembly at the end of mitosis does not proceed evenly around its surface but occurs differently in distinct peripheral regions (Liu and Pellman, 2020). When the process begins, the mitotic spindle is still in place, and in the area of overlap between it and chromatin (termed core region) the recruitment of some NE proteins is hampered. These proteins, including Nups, are initially recruited preferentially to the non-core region, where the spindle is not positioned (Kutay et al., 2021). Other proteins, including BAF, Emerin and LEM2), are initially enriched in the core region (Haraguchi et al., 2001; Dechat et al., 2004) (Figure 9A).

The factors that dictate the differential recruitment of NE proteins in the core vs. non-core regions are unclear. Evidence indicates that the mitotic spindle hampers the recruitment of non-core proteins such as Nups to the reforming NE at the core region (Liu et al., 2018). How this occurs is unknown. It has been proposed that ER sheets that bring the bulk of non-core proteins to the NE are too large to penetrate the spindle-occupied core region (Liu and Pellman, 2020). It is also possible that differences in kinase/phosphatase activity ratios between core and non-core regions impact the recruitment dynamics of some proteins. Mitotic kinases including Aurora B, Cyclin B-CDK1 and PLK1 are moderately enriched on the spindle until telophase, and they could locally compete with phosphatase to keep non-core proteins phosphorylated, preventing their recruitment (Afonso et al., 2019; Archambault and Glover, 2009; D'Avino and Capalbo, 2015). In addition, it has been hypothesized that NPCs assembling in the non-core region could promote the import of VRK1, which could keep BAF phosphorylated locally, inhibiting its recruitment (Liu and Pellman, 2020). At the core region, dephosphorylated BAF is readily recruited despite the presence of the spindle, possibly because BAF is not associated with membranes and because it avidly binds DNA. Once recruited at the core, BAF enables the enrichment of its LEM-Domain protein partners. Some of them are transmembrane proteins such as Emerin or LEM2, and we hypothesize that the core-enriched pool of these proteins is associated with membrane structures sufficiently small to infiltrate the spindle. Once recruited, LEM2 brings in ESCRT-III as presented above (von Appen et al., 2020). In addition to sealing new membranes, ESCRT-III and its partners CHMP7 and CC2D1B, promote the timely recruitment of Spastin, a MT-severing enzyme that participates in the disassembly of the spindle in the nascent nucleus before membranes can be sealed (Vietri et al., 2015; Ventimiglia et al., 2018). Therefore, the spatial division of core vs. non-core domains during the initial phase of NER may not be only a mere epiphenomenon as it may serve a cellular function in NR (Liu and Pellman, 2020).

Molecular mechanisms described above contribute to ensuring that the NE reassembles around chromosomes rather than at distant sites on ER (peripheral regulation). However, the cell also ensures that NER occurs on chromosomes after their segregation towards the poles (longitudinal regulation) (Figure 9B). In this way, lagging chromosomes tend not to assemble NE until they have rejoined the main chromosome mass (Afonso et al., 2014). This spatial regulation depends on the Aurora B kinase, which is part of the CPC. The CPC is concentrated at centromeres in prometaphase/metaphase and it re-localizes to the equatorial region of the central spindle in anaphase, remaining on the midbody arms in cytokinesis (Carmena et al., 2012b; D'Avino and Capalbo, 2015). As a result, Aurora B activity exists as a decreasing gradient from the equator to the poles throughout mitosis (Fuller et al., 2008; Wang et al., 2011; Afonso et al., 2014). This gradient inhibits the recruitment of NE proteins on chromosomes that have not completed their segregation on the spindle (Afonso et al., 2014). It likely also allows the correction of merotelic KT attachments of lagging chromosomes in anaphase (Cimini, 2007; Maiato et al., 2015). Since Aurora B promotes mitotic chromosome condensation, its gradient during mitotic exit may additionally prevent premature decondensation of lagging chromosomes (Maiato et al., 2015; Afonso et al., 2017). However, the specific contributions of the Aurora B gradient are difficult to untangle as decondensation and NER are closely coupled (Schellhaus et al., 2016). Through these mechanisms, the Aurora B kinase gradient was proposed to act as ruler that allows NR only on chromosomes that have segregated far enough, a control mechanism acting in parallel to the Cyclin-CDK dependent clock (Afonso et al., 2014). In addition, it was recently shown that the Aurora B gradient promotes the retention of a pool of Cyclin B-CDK1 on the central spindle, which could contribute to locally preventing NR events inhibited by CDK phosphorylation (Afonso et al., 2019).

An Aurora B-dependent mechanism also allows chromosome bridges to be resolved in anaphase before NER is completed. Genetically induced acentric chromosomes in *Drosophila* maintain tethers to the main mitotic chromosome mass as they trail behind during anaphase, thus generating chromosome bridges until they reintegrate the reassembling nucleus (Royou et al., 2010). These bridges undergo an Aurora B-dependent delay in NER as they maintain a channel with the main reassembling nucleus (Karg et al., 2015). This mechanism involves the concentration of Aurora B on chromatin bridges. Interestingly, Polo and BubR1 kinases are also present on the chromatin tethers (Royou et al., 2010). It remains to be determined if this regulation also operates in vertebrates.

Failure in these Aurora B-dependent mechanisms allows premature NER on lagging or bridging chromosomes and markedly increases the incidence of micronucleation (Warecki and Sullivan, 2018; Orr et al., 2021). It is assumed that these micronuclei assemble through the PP1 and PP2A-dependent NR-promoting mechanisms described in this review. Their relevant targets likely include substrates of Aurora B and CDK1, but could also include targets of Polo/PLK1, as it is activated by Aurora B and also enriched on lagging and bridging chromosomes (Royou et al., 2010; Carmena et al., 2012a; Kachaner et al., 2014; Karg et al., 2015).

## Conclusion and perspectives

The reformation of the nucleus at the end of an open mitosis presents a daunting challenge for the cell. While the dismantling of the interphase nucleus required for mitosis is induced by kinases that phosphorylate structural proteins to disrupt their interactions, the reassembly of the nucleus depends on phosphatases that target many of the same substrates to promote these interactions. This general principle largely accounts for the disassembly and reassembly of the NE and structures that govern chromatin organization. However, nuclear structures that are assembled in mitosis and disassembled during mitotic exit respond in the opposite way to the kinase/phosphatase cycle. This is the case for KT assembly and chromosome condensation, where phosphorylation events often promote protein interactions indirectly, by inducing a permissive conformation change or by blocking another post-translational modification that inhibits an interaction.

The precise dephosphorylation events required for NR, their mechanistic consequences and the identity of the phosphatases required for each event are not comprehensively understood. While NE reassembly is partially understood in those terms, less is known about chromosome decondensation. After surveying the functions of particular phosphatases in NR events, it becomes clear that PP1 enzymes, with multiple regulatory subunits, play a central role. PP2A enzymes also fulfill essential functions, although they are not as numerous as those of PP1. Additional functions of these and other phosphatases will almost surely emerge in the coming years. The decondensation and organization of chromatin within the nucleus during mitotic exit are bound to be facilitated in multiple ways by the progressive establishment of a nuclear environment that is biochemically distinct from the cytoplasm. This process involved the nuclear import and export of multiple enzymes and chromatin regulators, many of which are regulated by reversible phosphorylation in mitosis. Much research remains to be done to better understand these mechanisms that could impact NR in subtle or indirect ways.

The spatial and temporal coordination of NR events requires complex mechanisms, many of which have been uncovered in the last decade. These mechanisms consist of interconnections between mitotic kinases, phosphatases and some of their substrates, resulting in the timely and, in many cases, localized regulation of specific effectors in the mitotic cycle. While this review described mainly how mechanisms operate in human cells, this knowledge stems in large part from findings originally made in other model systems. Pursuing the exploration of mitosis in model organisms is guaranteed to accelerate discoveries of new mechanisms. Some of them will be conserved in humans while others will reveal interesting evolutionary plasticity.
